# Medical Society of the County of Genesee

**Published:** 1906-05

**Authors:** John A. Rafter


### Medical Society of the County of Genesee

Reported By JOHN A. RAFTER, M. D.

The Medical Society of the County of Genesee held its regular meeting in the court house at Batavia, April 4, 1906. Dr. H. E. Ganard presided and Dr. G. W. Cottis served as secretary. The members present were Drs. Tozier, Whitcomb, Snow, Spofford, Cottis, Johnson, A. F. Miller, Gardiner, and Manchester of Batavia; Ganard of Stafford; R. M. Andrews of Bergen; Prince, of Byron; Skinner and MacPherson of Le Roy; Hummel of Darien Center; Jackson of Oakfield; and J. B. Miller of Alexander. Drs. A. B. Miller of Syracuse, and Rochester and Meissenbach of Buffalo, were present as guests of the Society.

After routine business was concluded, Dr. A. B. Miller, a prominent surgeon of Syracuse, read a paper on "Surgical Treatment of Fibroids." Dr. Miller's paper was a strong, conservative and able presentation of the subject. Drs. Johnson, Cottis, Whitcomb and MacPherson discussed the paper. A vote of thanks of the society was tendered the author.

Dr. Hummel, of Darien Center, gave a report of a case of pelvic abscess. Dr. Alpheus Prince, of Byron, was elected delegate to the branch Medical Society of the 8th Judicial District.

